# Effects of Polylactide Copolymer Implants and Platelet-Rich Plasma on Bone Regeneration within a Large Calvarial Defect in Sheep

**DOI:** 10.1155/2018/4120471

**Published:** 2018-05-13

**Authors:** Bartłomiej Błaszczyk, Wojciech Kaspera, Krzysztof Ficek, Maciej Kajor, Marcin Binkowski, Ewa Stodolak-Zych, Aniela Grajoszek, Jerzy Stojko, Henryk Bursig, Piotr Ładziński

**Affiliations:** ^1^Department of Neurosurgery in Sosnowiec, Medical University of Silesia, Katowice, Poland; ^2^Academy of Physical Education, Katowice, Poland; ^3^Department of Pathology, Medical University of Silesia, Katowice, Poland; ^4^Department of Biomedical Computer System, Faculty of Computer and Materials Science, University of Silesia, Chorzow, Poland; ^5^Department of Biomaterials, Faculty of Materials Science and Ceramics, University of Science and Technology, Krakow, Poland; ^6^Department for Experimental Medicine, Medical University of Silesia, Katowice, Poland; ^7^Regional Blood Center, Katowice, Poland

## Abstract

The aim of this study was to verify whether L-lactide/DL-lactide copolymer 80/20 (PLDLLA) and platelet-rich plasma (PRP) trigger bone formation within critical-sized calvarial defects in adult sheep (*n* = 6). Two craniectomies, each ca. 3 cm in diameter, were created in each animal. The first craniectomy was protected with an inner polylactide membrane, filled with PRP-polylactide granules, and covered with outer polylactide membrane. The second control craniectomy was left untreated. The animals were euthanized at 6, 7, 17, 19, 33, and 34 weeks after surgery, and the quality and the rate of reossification were assessed histomorphometrically and microtomographically. The study demonstrated that application of implants made of PLDLLA 80/20 combined with an osteopromotive substance (e.g., PRP) may promote bone healing in large calvarial defect in sheep. These promising proof-of-concept studies need to be verified in the future on a larger cohort of animals and over a longer period of time in order to draw definitive conclusions.

## 1. Introduction

Skull bone reconstruction is frequently performed within departments of neurosurgery as some neurosurgical procedures require craniectomy. Restoration of skull integrity is a vital procedure, as it protects fragile neural structures against injury, provides appropriate dynamics of cerebrospinal flow, and prevents ischemia caused by herniation of the brain into the craniectomy. Skull defects can be repaired with autologous bone grafts or synthetic materials. The most popular synthetic nonresorbable material is polymethyl methacrylate (PMMA) used as bone cement (i.e., polymerized in situ from methyl methacrylate monomer and polymer powder mixture), as well as a bulk polymer implant material. PMMA is considered a reliable and inexpensive implant material, but it may cause local toxicity and become encapsulated by fibrous tissue. Furthermore, nonresorbable synthetic materials pose a 10–40% risk of infection or graft rejection [1, 2]. In turn, the use of frozen autologous bone grafts, aside from a 25% risk of infection, is also associated with up to 15% risk of bone flap resorption [3]. Therefore, against this background, novel alternatives for autologous grafts and nonresorbable synthetic materials are urgently required. Ideally, it should be synthetic resorbable material or nanocomposite (polymer-ceramic) based on biodegradable polymer scaffolds and bioactive ceramic fillers [4–11]. One of the most promising materials so far are polylactide copolymers and several groups have already demonstrated their osteogenic properties and capabilities of healing bone defects [12–20]. Polylactide copolymers exist as two isomeric forms, L-polylactide and D-polylactide. Polymerization of a racemic L-lactide and D-lactide mixture results in synthesis of amorphous poly-DL-lactide. Up to date, two forms of the copolymer, with 80% to 20% and 70% to 30% L-lactide to DL-lactide ratio, have found application in experimental and clinical studies. Of note, the first has greater strength, better dimensional stability, and higher degree of crystallinity and biodegradability [14, 21].

Even though the previous studies have demonstrated usefulness of polylactide polymers in reconstruction of small bone defects, their applicability in the treatment of larger defects still remains an open question. This might be due to the fact that their osteoconductive and osteoinductive properties are not as good as those of autologous bones.

As a result, resorption of a synthetic material used to fill the defect is not counterbalanced by the synthesis of the new bone, which may eventually lead to pathological fractures or nonintegration in the vicinity of the graft [22]. Consequently, synthetic reconstructive materials should be applied together with osteoinductive/osteogenic materials, such as platelet-rich plasma (PRP) and its derivatives (PRGF, plasma-rich growth factors; PRF, platelet-rich fibrin) or mesenchymal stem cells derived from bone marrow/connective/adipose/dental tissues [23–27]. Several animal and human studies demonstrated that bone marrow/adipose/dental-derived mesenchymal stem cells/PRF promote bone growth, due to the ability of mesenchymal cells to transform into osteoblasts or under the influence of growth factors released from platelets that may stimulate processes responsible for bone healing (e.g., mitogenesis, chemotaxis, and cell differentiation) [23–25, 28–31]. Both PRP and its derivatives/mesenchymal cells are currently widely used as a stimulator of bone healing during reconstructive procedures in maxillofacial surgery [23–25, 32–34].

Herein, we present the proof-of-concept experiment that focuses on regeneration of large bone defects using synthetic materials with established osteoconductive properties (e.g., polylactide and calcium triphosphate), in a form of composite, along with osteoinductive substances, such as bone marrow and/or PRP. The aim of this study was to investigate the process of bone formation within L-lactide/DL-lactide copolymer 80/20 (PLDLLA) and PRP-filled critical calvarial defects in a medium-sized animal (sheep).

## 2. Materials and Methods

### 2.1. Animals

The experiment was performed using six adult female Merino sheep* (Ovis aries)*, aged approximately three years, with 70–80 kg body weight. The animals were kept in an appropriately adapted room and fed with a standard laboratory diet. The protocol of the study was approved by the Animal Research Ethics Committee at the Medical University of Silesia (decision number 55/2010).

### 2.2. Implants

A commercially available FDA-attested basic 80/20 PLDLLA polymer (PURAC Biochem, Gorinchem, Netherlands) with molecular weight of ca. 200 kDa was used. Neat polymer was processed and tested in two forms, membrane and porous granules. Thin (350 *μ*m) polymer membranes were obtained by a 10 min hot pressing of polymer beads in a stainless form (4 × 12 cm) at 160°C.

Topography of the membrane surface was examined goniometrically (HOMMEL TESTER T500, Germany). The results are expressed as mean surface roughness (*R*_*a*_) determined during 10 repeated measurements, along with standard deviation (SD). Wettability (Θ) of the composite surface was determined directly (DSA 10, Krüss GmbH, Germany). The measurements were taken at room temperature (RT), using high-purity water (PURELAB UHQ, Vivendi Water, UK) as a measuring liquid. Microstructure of the membrane was examined using a scanning electron microscope (Nova NanoSEM, FEI, USA). Mechanical parameters of the membrane, such as tensile strength (*R*_*m*_) and Young's modulus (*E*), were measured with a tensile tester machine (Zwick 1435, Zwick Roell, Germany), with a parallel specimen length and test speed of 40 mm and 40 mm/min, respectively. Six measurements were taken for each sample. The results of the measurements are summarized in Table 1.

**Figure 1:**
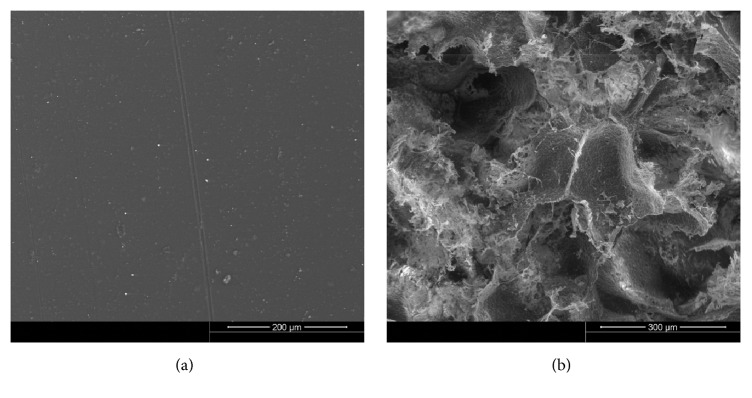
SEM microphotographs of polylactide membrane surface (a) and porous polylactide granule (b).

Porous polymer granules with PLDLLA were obtained using solvent casting and a specific leaching method, with sodium dihydrogen phosphate hexahydrate (NaH_2_PO_4_·6H_2_O, Avantor S.A., Poland) as a porogene. Two fractions of slat particles, 100 *μ*m and 300 *μ*m at 1 : 2 ratio, were used during the preparation process. During the first step, PLDLLA was dissolved in acetone (CH_3_COCH_3_, Avantor S.A., Poland; 1 : 6 polymer to solvent ratio) at RT for 24 h. Then the solution was mixed with salt particles at 1 : 35 ratio (dry mass of PLDLLA to NaH_2_PO_4_·6H_2_O content) with the aid of a mechanical stirrer (30°C/15 min). The mixture was air-dried for 72 h at 25°C and then transferred to a vacuum chamber at 25°C for 24 h. Then, the salt particles were dissolved in water (20°C) until ion conductivity of the water bath contents, monitored with pH-meter (CP400, Elmetron, Poland), reached that of pure water (i.e., 3.5 *μ*m/cm). Only the granules with 100 to 400 *μ*m diameters were chosen for further experiments. Dimensions (*d*) of the porous granules and their morphological characteristics were determined with Mastersizer 2000 (Malvern Instruments Ltd., UK) and Nova NanoSEM (FEI, USA), respectively. Total porosity (*P*) and pore size (*p*) were measured by means of mercury porosimetry (PoreMaster 60, Quantachrome Instruments, USA). Also, specific surface area (*σ*_m_) of the granules was determined. All the above-mentioned parameters of porous granules are presented in Table 2 and Figure 1.

### 2.3. PRP Preparation

A few minutes before introduction of anesthesia, 25 ml blood sample was obtained from the external jugular vein of each animal. The samples were collected to silicone vials with 0.5 ml 3.2% sodium citrate and centrifuged at 2,400 rpm for 10 min (MPW-341 centrifuge, MPW Med. Instruments, Warsaw, Poland). As a result, three layers of blood components were obtained: the bottom layer containing erythrocytes, a top layer of plasma with a trace number of platelets, that is, the so-called platelet-poor plasma (PPP), and a white intermediate layer of PRP with a trace amount of leukocytes. Then, the layer of PPP was pipetted to new vials and further centrifuged at 5,000 rpm for 10 min. After pooling PRP from the first and second centrifugation, ca. 1.5 ml aliquots were obtained with 2.4-fold greater mean platelet density than in the source whole blood sample. Upon adding ca. 20 *μ*L 10% calcium chloride solution (CaCl_2_, Avantor S.A., Poland) to activate the platelets, PRP was mixed with polylactide granules to form a gel that was used during surgical procedures. Amount of the granules was about 400 *μ*g, while volume of the polymer granules mixed with PRP and tightly packed during craniotomy was about 3.5 ml.

### 2.4. Surgical Procedure

Peripheral catheters were inserted into both external jugular veins of each animal. Anesthesia was introduced with atropine (1 mg, Atropinum Sulfuricum 1 mg/ml injection, Polfa Warszawa, Poland) and ketamine (1 mg per kg body weight, usually 70 mg, Ketanest 50 mg/ml, Pfizer Europe, UK), followed by thiopental (1 g dissolved in 20 ml 0.9% NaCl, Thiopental Injection BP 500 mg, Rotexmedica GmbH, Germany) and fentanyl (0.1 mg, Fentanyl WZF, 50 *μ*g/ml injection, Polfa Warszawa, Poland). Then, the animal was intubated with the aid of a laryngoscope, an inhalation anesthetic device was connected to the tracheal tube, and general inhalation anesthesia with isoflurane (Aerrane 250 ml, Baxter, Poland) was introduced gradually, initially at 2%, and lowered to 1–1.5% concentration. A single intravenous injection of cefazolin (1 g, Biofazolin for Injection USP 1 g, Polpharma, Poland) was given as a perioperative antibiotic prophylaxis.

**Figure 2:**
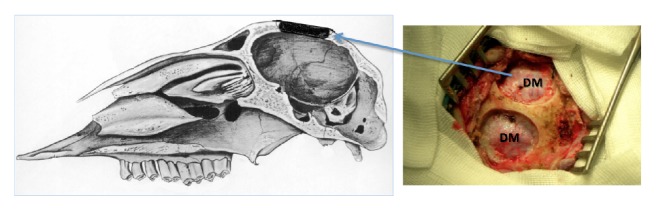
Sagittal cross section of ovine skull and intraoperative photograph of two calvarial craniectomies. DM: dura mater.

After preparation of the surgical field, a T-shaped skin incision was made, with a transverse incision along the biauricular line and shorter midline incision oriented caudally. Then, a cutaneoaponeurotic flap was separated from the calvarium with a monopolar electric knife, and two craniectomies, each ca. 3 cm in diameter, were made in the right and left frontoparietal regions, next to the sagittal line, without compromising the dura mater (Figure 2). Due to specific anatomical conditions of calvarium in adult female sheep, we were unable to create more than two critical openings. One craniectomy in each animal was left empty as the control, whereas the second one was filled with the polylactide porous granules and PRP as described below.

**Figure 3:**
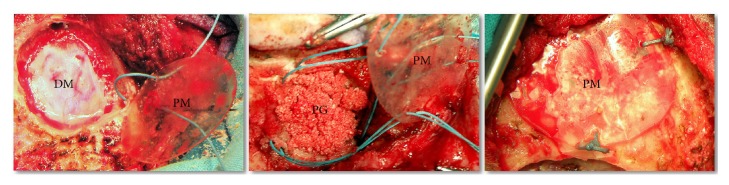
Consecutive stages of filling experimental craniectomy with polylactide implants and platelet-rich plasma (PRP). DM: dura mater; PG: polylactide granules mixed with PRP; PM: polylactide membrane.

One of the polylactide membranes, adjusted for the size and shape of the opening, was placed at the craniectomy bottom. Then, the defect was filled with the gel composed of PRP and polylactide granules and covered with another polylactide membrane, with the diameter being 5 mm larger than that of the craniectomy opening. The outer membrane was fixed to the bone at three points, with sutures placed in previously drilled small holes. The procedure of filling the defect with polylactide is presented schematically on Figure 3.

During the last stage of the procedure, after achieving hemostasis, the skin wound was closed with multiple layers of stitches, and a hydrocolloid gel with stabilized silver complex (Hydrosil-Flamozil, Sequoia Sp. z o.o., Poland) was applied to prevent bacterial infection and to facilitate healing.

After the procedure, all animals were kept under the same conditions as preoperatively and fed with a standard laboratory diet. None of the animals presented with fever or signs of infection. Skin stitches were removed 7 days after surgery. First intention healing was achieved in all cases. To analyze the influence of time on bone regeneration, the animals were euthanized consecutively at 6, 7, 17, 19, 33, and 34 weeks after surgery; a ketamine injection (1 mg per kg body weight) was given followed by propofol infusion (200–400 mg, Propofol 1% Fresenius, Fresenius Kabi Deutschland GmbH, Germany) to cause loss of consciousness, respiratory depression, and death.

### 2.5. Microscopic Analysis and Histomorphometry

A fragment of the calvarium containing both primary and newly formed bone from the regeneration site was obtained from each animal. The specimens were transferred to the Department of Pathology, Medical University of Silesia in Katowice, whereby thin slices were cut from peripheral and central portion of the regeneration area. The slices were fixed in a 1 : 1 mixture of 10% neutral buffered formalin (Bio-Optica Milano S.p.A., Milan, Italy) and 10% aqueous EDTA solution (tetrasodium versenate hydrate, Avantor S.A., Poland). After decalcification, slices were rinsed with 70% ethanol and cut into 5 *μ*m sections using a microtome (Hyrax M55, Zeiss, Germany). Microscopic specimens were stained with Masson's trichrome. A total of 10 microscopic specimens were prepared for each animal: 5 from the central portion and 5 from the peripheral portion of the regeneration site. The specimens were examined at 200x under an Olympus BX53 Digital Upright Microscope (Olympus Deutschland GmbH, Hamburg, Germany) and representative digital images were analyzed by Image-Pro Plus 6.0 software (Media Cybernetics, Inc., Rockville, USA). The total surface area of all five specimens was 738,840 *μ*m^2^. Computerized analysis included identification and quantification of three components: bone trabeculae, immature bone, and connective tissue. Bone trabeculae and the foci of immature bone tissue were quantified in both the peripheral and central portions of each specimen; specimen areas occupied by these components were determined, along with the area occupied by the connective tissue.

### 2.6. Microtomographic Analysis

During the next stage, bone density was determined in the calvarium specimens. Cuboid samples with dimensions adjusted to the skull bone thickness in a given animal and mean volume of 350 mm^3^ were obtained from each specimen and examined using a high-resolution micro-CT scanner (phoenix v∣tome∣x s, General Electric Measurement & Control Solutions, Wunstorf, Germany). Global thresholding at various levels was performed in order to separately visualize soft and hard tissues by segmentation.

### 2.7. Statistical Analysis

Statistical significance of differences in the quality and rate of tissue reconstruction in central and peripheral regions of the defect was verified with the nonparametric Wilcoxon signed-rank test. Associations between follow-up time and the area of newly formed bone and between the latter parameter and hydroxyapatite (HAp) density were studied on the basis of Spearman's rank correlation coefficient. Total areas occupied by newly formed bone and connective tissue in specimens from two animals euthanized at 6 and 7, 17 and 19, and 33 and 34 weeks were compared with the Kruskal-Wallis test. All calculations were carried out with STATISTICA™ 10 (StatSoft, Inc. (2011), http://www.statsoft.com), with the threshold of statistical significance set at *p* < 0.05.

## 3. Results

### 3.1. Macroscopic Analysis and Imaging

As expected, there were no signs of ossification in any of the control craniectomies. All of them were filled with connective tissue, tightly attached to the dura. Moreover, we found that their bone edges underwent remodeling and resorption becoming more rounded.

**Figure 4:**
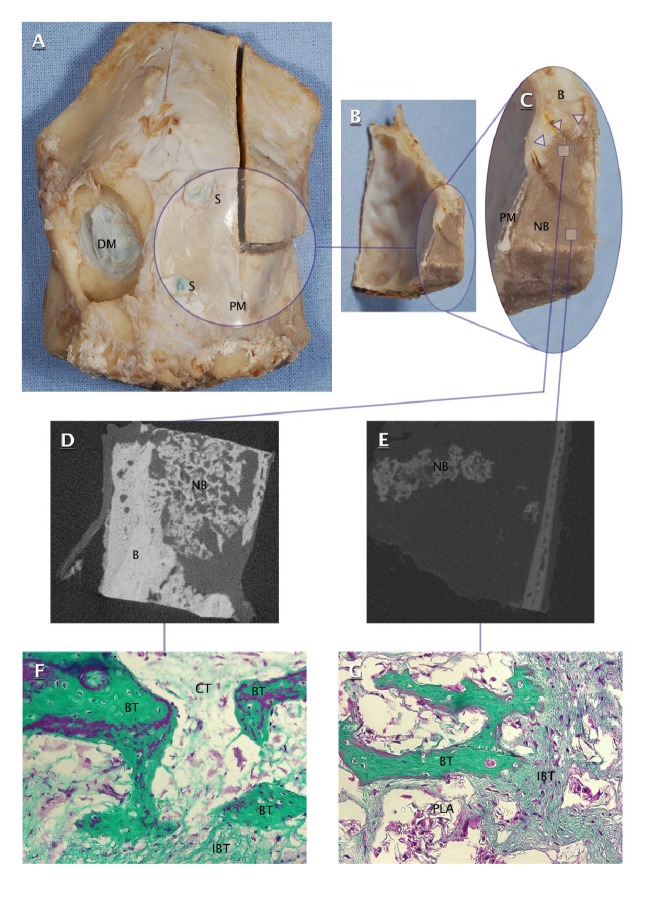
Ovine calvarial specimen (A), transverse cross sections of filled craniectomy ((B) and (C)), microtomographic images of peripheral part (D) and central part (E) of filled craniectomy, and microphotographs of peripheral part (F) and central part (G) of filled craniectomy (200x, Masson's trichrome). DM: dura mater; S: suture; PM: polylactide membrane; B: calvarial bone; NB: newly formed bone in filled craniectomy; Δ: interface of newly formed bone and craniectomy edge; BT: bone trabecula; IBT: immature bone tissue; CT: connective tissue; PLA: polylactide.

On the other hand, craniectomies filled with polylactide/PRP gel showed the presence of irregular osteoid tissue, slightly darker than surrounding primary bone (Figures 4(A), 4(B), and 4(C)). This osteoid tissue was identified as a mixture of bone, connective tissue, and polylactide residues. The resultant conglomerate adhered tightly to the bone edges of craniectomy and kept a stable position within the defect. Importantly, both polylactide membranes retained their morphological integrity and showed no signs of degradation. Furthermore, the microtomographic studies revealed the areas filled with mineralized bone. Some of these areas were in direct contact with the bone edges of craniectomy, whereas others appeared as islands of bone tissue spread within central parts of the defect (Figures 4(D) and 4(E)).

### 3.2. Histological and Microtomographic Analysis

Histological analysis of specimens from craniectomies filled with polylactide/PRP gel included 60 specimens stained with Masson's method, 10 per animal including 5 from the central portion and 5 from the periphery of the defect. No signs of inflammation associated with the infection or presence of a foreign body were found in histological specimens. Representative microscopic images are presented on Figures 4(f) and 4(g). Individual data for microtomographically determined HAp density and histomorphometrically determined areas of ossification foci (i.e., bone trabeculae and nontrabecular bone) and connective tissue are presented in Table 3.

**Figure 5:**
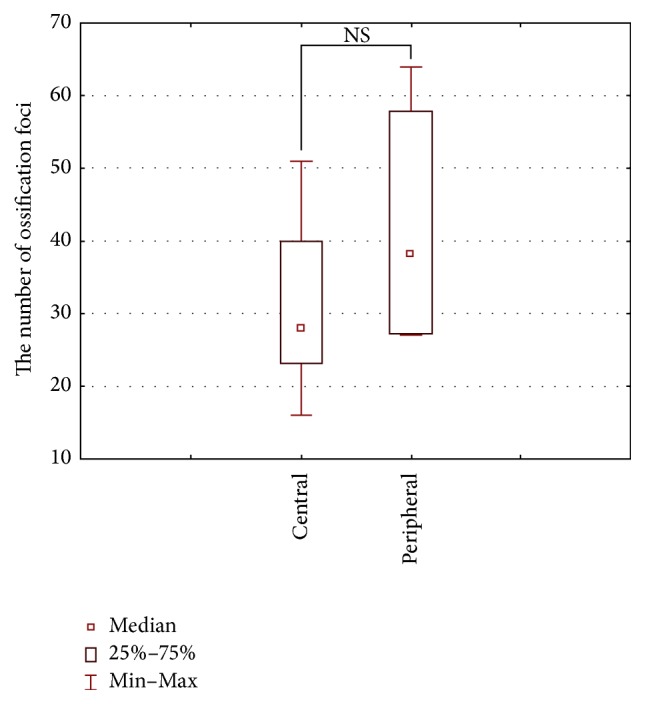
Number of ossification foci (the sum of bone trabeculae and immature bone tissue) in the peripheral and central parts of the defect (Wilcoxon signed-rank test).

**Figure 6:**
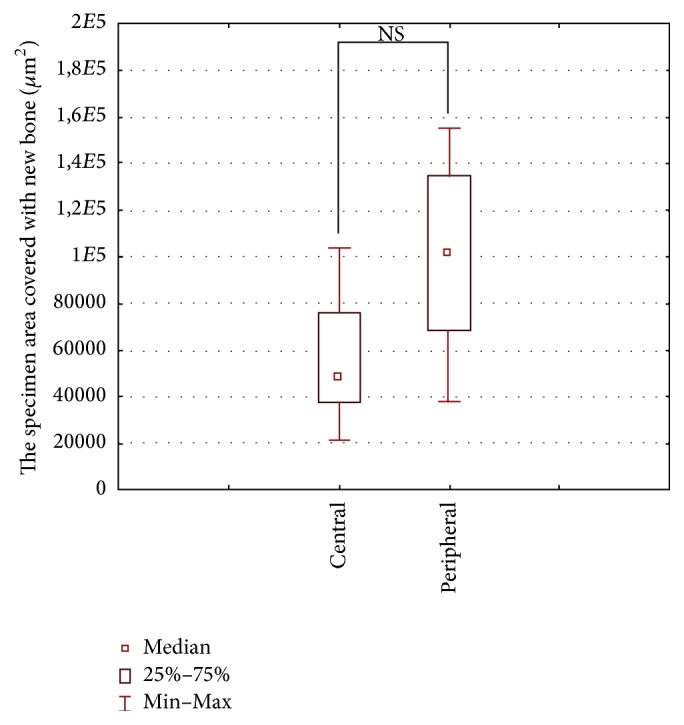
Area covered by newly formed bone (the sum of bone trabeculae and immature bone tissue) in the peripheral and central parts of the defect (Wilcoxon signed-rank test).

**Figure 7:**
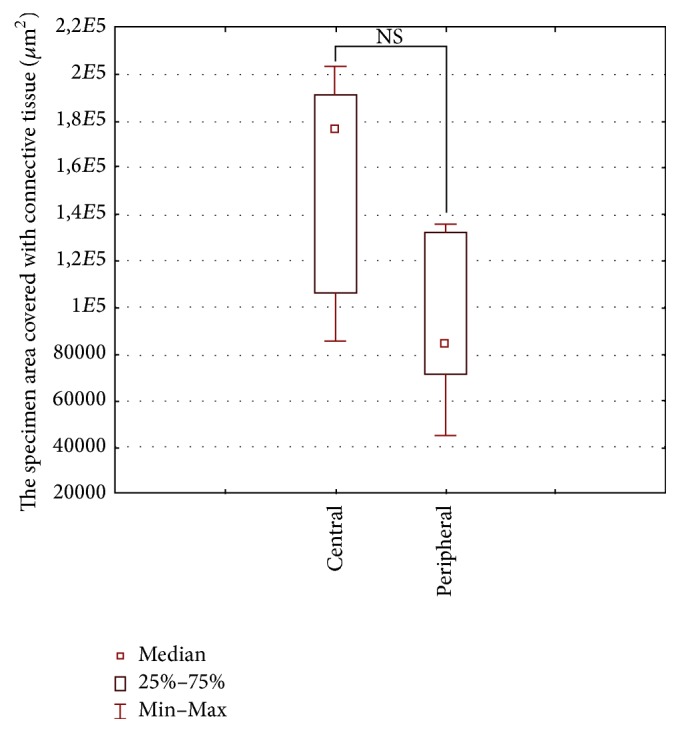
Area covered by connective tissue in the peripheral and central parts of the defect (Wilcoxon signed-rank test).

Median values (along with the 25th and 75th percentiles) for the number and area of ossification foci and the specimen area covered with connective tissue for the central part of the defect were 28 (24–37), 48 × 10^3^ (38 × 10^3^–76 × 10^3^) *μ*m^2^, and 176 × 10^3^ (120 × 10^3^–190 × 10^3^) *μ*m^2^, and for its peripheral portion 38 (27–55), 102 × 10^3^ (69 × 10^3^–136 × 10^3^) *μ*m^2^, and 84 × 10^3^ (71 × 10^3^–123 × 10^3^) *μ*m^2^, respectively. Central and peripheral regions of the defect did not differ significantly in terms of the number and the area of ossification foci and the specimen area covered with connective tissue (Figures 5–7).

When the rate of tissue regeneration was stratified according to observation time, mean amount of bone tissue amounted to 119 × 10^3^ *μ*m^2^ (i.e., 8% of histological specimen surface) for 6 and 7 weeks, 143 × 10^3^ *μ*m^2^ (i.e., 9.8%) for 17 and 19 weeks, and 201 × 10^3^ *μ*m^2^ (i.e., 13.6%) for 33 and 34 weeks, whereas mean contents of connective tissue were 193 × 10^3^ *μ*m^2^ (i.e., 13.1%), 238 × 10^3^ *μ*m^2^ (i.e., 16.1%), and 313 × 10^3^ *μ*m^2^ (21.1%), respectively (Table 3). In turn, mean contents of bone tissue and connective tissue for all histological specimens, irrespective of the observation time, were 154 × 10^3^ *μ*m^2^ (10.5%) and 248 × 10^3^ *μ*m^2^ (16.8%), respectively.

**Figure 8:**
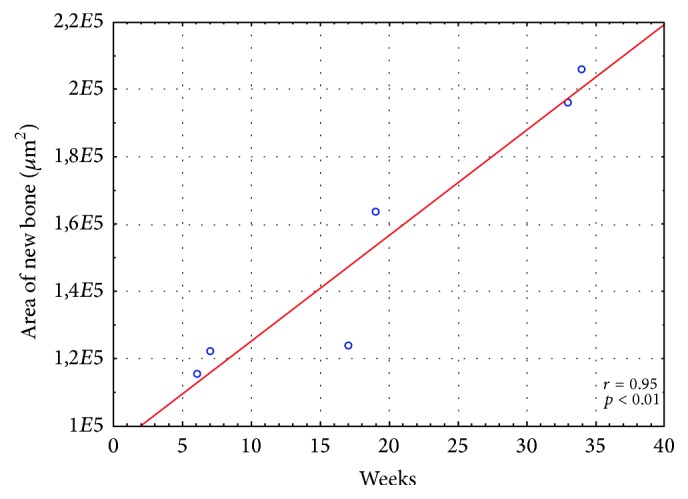
Correlation between follow-up time and total specimen area covered with newly formed bone (Spearman's rank correlation coefficient).

A significant positive correlation was found between total area covered with newly formed bone and HAp density (*r* = 0.94; *p* < 0.01) (Table 3). Moreover, a strong correlation (*r* = 0.95; *p* < 0.01) was found between the area of ossification foci in analyzed microscopic specimens and follow-up time in weeks (Figure 8). There were no statistically significant differences in the areas covered with newly formed bone and connective tissue in specimens from 6 and 7, 17 and 19, and 33 and 34 weeks of follow-up (Table 3). This implies that, after an initial intensive regeneration of bone tissue, this process still progressed but at a substantially lower rate.

## 4. Discussion

The important parameters in the biocompatibility study of each biomaterial, including polymers, are their surface and their impact on the regeneration processes after implantation [35]. The cell-biomaterial interactions are influenced by surface morphology (porosity and roughness) and other features of the surface layers (e.g., chemical composition and physicochemical properties) which depend on the polymer processing method (thermal history and additional physical, chemical, or biological treatment). In order to develop a high-quality biocompatible biomaterial, several sample features need to be initially tested and standardized* in vitro*. For example, Marrelli et al. have developed a computer-aided design (CAD) project that used a standardized design to test different types of scaffolds in order to provide a greater accuracy of the measurements in the assessment of the cellular response on a biomaterial [36]. Our* in vivo* experiments were preceded by* in vitro* tests that confirmed good biocompatibility of the porous form of PLDLLA-based biomaterial with osteoblasts [37, 38]. Furthermore, we have also demonstrated previously that both porosity and morphology of granules and membranes are beneficial for the safe transfer of PRP elements (i.e., growth factors) [39].

The current* in vivo* experiment has shown, in line with the previous findings [12, 14, 15, 17–19], that poly(L/DL-lactide) 80/20-based implants are biocompatible and well tolerated by host tissues. We observed no signs of adverse tissue reactions such as infiltration of inflammatory cells or formation of necrotic foci in the examined specimens.

In addition, we demonstrated that L/DL 80/20 polylactide-based implants promote bone regeneration within medium-sized skull defects. We found ossification foci in both the peripheral and central portions of the defects already 6 weeks after implantation. During following weeks, the amount of newly formed bone (bone trabeculae and immature bone matrix) increased linearly (Figure 8), suggesting that osteoinductive activity of PRP might have been overlapped with an osteoconductive effect of porous polylactide granules.

We did not find any significant differences in the amount of bone trabeculae and immature bone matrix in the peripheral and central parts of the defect (Figures 5 and 6). However, it is well known that ossification begins at the periphery of the defect and progresses towards its central part, which is due to the fact that periosteum and bone marrow are a primary source for osteogenic cells during regeneration. Our findings imply that the rates of bone regeneration in the center and periphery of the defect were essentially the same. Most likely, osteogenesis in the central part of the defect was additionally stimulated by osteogenic cells derived from dura mater or by the connective tissue cells penetrating across the barrier membrane and differentiating into osseous tissue. Of note, it has been previously shown that connective tissue cells may undergo transformation into osseous tissue, especially the osteogenic factors that are released from the bone ends and/or bone marrow present in the defect [18].

The histomorphometric analysis has shown that, at the end of the experiment, in less than a year (at week 34), newly formed ossification foci covered merely ca. 14% of the defect (Table 3). However, the microtomographic analysis provided more optimistic results, showing that the level of HAp, an inorganic component of bone, reached up to 76% (Table 3). High concentration of HAp within the defect most likely resulted from the mineralization of the latter, stimulated by addition of osteoinductive PRP. This indicates that the tissue growing on the scaffold of polylactide granules enriched with PRP may show similar durability to normal bone, providing adequate regeneration of large skull defects. These results are in line with data published by other authors who used scaffolds made of ceramic biomaterials, such as HAp and calcium triphosphate [6, 10, 11].

Although the differences in the amount of new bone at three examined time points were not statistically significant (Table 3), we found a strong correlation (*r* = 0.95; *p* < 0.01) between the area of ossification foci in histological specimens and observation time expressed in weeks (Figure 8). This suggests that although the rate of bone regeneration decreased after an initial peak, the process was still progressing. This observation is consistent with previously published studies, in which the amount of newly formed bone increased steadily with observation time, despite the lack of statistically significant differences between the analyzed time points [10–12, 15, 40].

A decrease in the rate of bone regeneration seems to be a consequence of connective tissue ingrowth into the bone defect. The technique of guided bone regeneration (GBR) with barrier membranes covering the defect plays a key role in the prevention of connective tissue ingrowth [12, 14, 15, 18, 19, 41–44]. The success of GBR technique depends on physical support provided by the barrier membrane that overlays the soft tissue and creates a space to be filled with blood clot while excluding the competing nonosteogenic cells from the defect and possibly allowing local accumulation of the growth factors under the membrane [18]. A number of authors reported successful application of GBR technique in the treatment of small bone defects [5, 7, 12, 13, 18, 43, 45], including small calvarial defects [14–16, 19, 40–42, 46]. In these studies, bone defects were covered with membranes made of resorbable materials with good biocompatibility: polylactide, polyglycolide, copolymers of lactide and glycolide [12–16, 18, 19, 42, 43], polyhydroxybutyrate acid [45] or nonresorbable polytetrafluoroethylene [41], calcium sulfate [5], and others [7, 46]. The most common type of polylactide copolymer-based implants used in animal studies was membranes made of poly(L-lactide), poly(D,L-lactide), and poly(L/DL-lactide) 70 : 30 and 80 : 20 [12, 14, 15, 18, 19, 43]. In the treatment of large bone defects aside from barrier membranes, also fillers with established osseointegration properties, *β*-tricalcium phosphate (*β*-TCP) [4, 6, 11], HAp [10, 47], biocompatible ceramic materials [5, 8, 9], and others [7, 48], can be used along with osteopromotive materials, such as bone marrow-derived mesenchymal stem cells and PRP. However, if the barrier membranes degrade too rapidly, they may lose their morphological integrity and physical strength and eventually collapse inside the defect [41, 46]. The advantage of polylactide copolymer-based membranes is relatively long degradation time, up to several months [12, 18, 49]. Furthermore, the differences in biodegradability between different types of the polylactide membranes do not seem to exert a significant effect on bone regeneration [14, 15, 50]. In our present study, although the membranes made of PLDLLA 80/20 retained their integrity and elasticity, still promoting the process of bone regeneration nearly a year after implantation, they did not prevent slowdown of bone regeneration and penetration of connective tissue into the defect (ca. 23% of connective tissue versus ca. 14% of newly formed bone covered the defect at the end of the experiment, Table 3). Probably, the reason behind infiltration of fibroblasts into the defect and the decrease in bone regeneration rate that we observed was imperfect fixation of the outer membrane to the bone. Improvement of fixation of the outer membrane may influence the rate of bone regeneration. For instance, Amano et al. [43] have demonstrated that, at the end of a 36-week follow-up, the percentage of new bone filling the space beneath the test membrane held by fixing pins was greater than beneath the nonfixed membrane (62% versus 53%; *p* < 0.05).

While the role of outer membrane in the process of bone regeneration is well established, the applicability of inner membrane placed between the dura and the inner surface of calvarial bone is still an open question. Importantly, the authors who used both outer and inner membranes observed higher rate of bone regeneration and better organization of newly formed bone trabeculae, which is a promising finding [14, 41, 42]. Both membranes act as a barrier, protecting the defect against connective tissue ingrowth and preventing prolapse of the dura. Moreover, the use of two membranes may be helpful in restoration of cranial curvature in the case of larger defects. However, application of inner membrane in our experiment may raise some questions, since, due to characteristic shape and relatively small size of ovine skull, artificially created bone defects were essentially flat and round, and tense intact dura retained desirable shape of the calvarium. Furthermore, histological analysis demonstrated that bone trabeculae and immature bone matrix formed primarily on the scaffold made of polylactide granules rather than on the membranes. Therefore, it cannot be excluded that the inner membrane did not promote bone regeneration but could have even interfered with the osteoinductive effect of the dura.

To sum up, our study have shown that implants made of poly(L/DL-lactide) 80/20 have satisfactory biocompatibility and most likely exert an osteoconductive effect together with osteoinductive PRP, promoting bone regeneration within large skull defects in sheep.

## 5. Conclusions

Implantation of poly(L/DL-lactide) 80/20 scaffolds in combination with PRP acts synergistically as poly(L/DL-lactide) 80/20 provides osteoconduction (due to porosity of PLDLLA implants) for the osteoinductivity of the PRP promoting bone regeneration within large calvarial defect. These promising, preliminary findings need to be verified in the future in a larger group of animals followed up over a longer period of time in order to draw definitive conclusions. In addition, various forms of PLDLLA, that is, porous granules as a PRP carrier and a membrane for defect closure, could be replaced in the healing of bone defects with another form of the membrane, such as a 3D-printed implant, which despite the porosity also has biomechanical strength to accelerate cell infiltration even after degradation of the material. Moreover, the fixation of an outer polylactide membrane to bone edges should be optimized to prevent connective tissue ingrowth into the defect. Further improvements of bone regeneration rate within large cranial defects might require application of composite fillers made of polylactide polymers and other materials with osseointegration properties, such as HAp or calcium phosphate.

## Figures and Tables

**Table 1:** Physicochemical and mechanical parameters of investigated membrane implants.

	Physicochemical parameters		Mechanical parameters	
	Wettability Θ [°]	Roughness *R*_*a*_ [*μ*m]	Tensile strength *R*_*m*_ [MPa]	Young's modulus *E* [GPa]
PLDLLA membrane	78.4 ± 2.53	1.52 ± 0.12	48.5 ± 2.98	2.32 ± 0.32

PLDLLA: L-lactide and DL-lactide copolymer (poly-L/DL-lactide).

**Table 2:** Microstructural and physical parameters of the porous granules.

	Microstructural parameters			Physical parameter
	Porosity, *P* [%]	Pore size, *p* [*μ*m]	Specific surface area, *σ*_*m*_ [m^2^/g]	Size of granules, *d* [*μ*m]
Porous granules	~75	5–200	2.1	100–400

**Table 3:** Comparison of histomorphometric and microtomographic parameters.

Animal number	Follow-up (weeks)	Absolute (*μ*m^2^) and relative (%) contents of new bone in a specimen^*∗∗*^	Absolute (*μ*m^2^) and relative (%) contents of connective tissue in a specimen^*∗∗*^	HAp density (g/cm^3^) and corresponding percentage of reference value^*∗*^
II	6	115 × 10^3^ (7.8%)	156 × 10^3^ (10.6%)	0.88 (58.7%)
V	7	122 × 10^3^ (8.3%)	231 × 10^3^ (15.6%)	0.97 (64.7%)
VI	17	123 × 10^3^ (8.4%)	238 × 10^3^ (16.1%)	1.02 (68.0%)
III	19	163 × 10^3^ (11.1%)	238 × 10^3^ (16.2%)	1.01 (67.3%)
IV	33	196 × 10^3^ (13.3%)	287 × 10^3^ (19.4%)	1.06 (70.7%)
I	34	206 × 10^3^ (13.9%)	339 × 10^3^ (22.9%)	1.14 (76.0%)

Data represent five specimens from the peripheral part and five specimens from the central part. HAp: hydroxyapatite. There is a significant positive correlation between total area covered with newly formed bone and hydroxyapatite density (Spearman's *r* = 0.94; *p* < 0.01). ^*∗*^HAp density in normal bone—1.50 g/cm^3^; ^*∗∗*^nonsignificant differences in histomorphometric findings determined at 6-7, 17–19, and 33-34 weeks (Kruskal-Wallis test).
